# Efficacy of mini-scleral lenses in visual rehabilitation for corneal disorders: a prospective study

**DOI:** 10.1186/s12886-025-04155-z

**Published:** 2025-05-31

**Authors:** Jaya Kaushik, Sumit Goyal, Ankita Singh, Vipin Rana, Jitendra Kumar Singh Parihar, Alok Sati, Mayank Jhanwar

**Affiliations:** 1 Dept of Ophthalmology, Command Hospital (CC), Lucknow, India; 2 Department of Ophthalmology, Military Hospital, Bareilly, India; 3 Command Hospital (EC), Kolkata, India; 4 Centre For Sight, New Delhi, India; 5 Dept of Ophthalmology, Armed Forces Medical Services, New Delhi, India; 6 Department of Ophthalmology, INHS Sanjivani, Kochi, India

**Keywords:** Mini-scleral lenses, Corneal irregularities, Keratoconus, Corneal scarring, Post-refractive surgery

## Abstract

**Background:**

Corneal irregularities are a significant cause of visual impairment, impacting patients’ quality of life. Conventional treatment with spectacles and/or contact lenses fail to provide adequate rehabilitation in these cases. This study aimed to evaluate the efficacy and patient satisfaction with use of mini-scleral devices (MSDs) in patients with various corneal disorders including keratoconus, corneal scarring, post-corneal transplant, and post-refractive surgery.

**Methods:**

This analytical, observational, prospective study included 40 eyes of 32 patients with corneal surface irregularities and rigid gas permeable (RGP) or soft contact lenses intolerance. Comprehensive ophthalmic evaluation, including corneal topography, was performed. Primary outcomes measured were improvement in best lens-corrected visual acuity (BLCVA) by ≥ 2 lines and daily wear time of ≥ 10 h. Secondary outcomes included patient comfort, lens acceptance, and adverse events. Patients were followed up for a duration of three months.

**Results:**

MSDs significantly improved visual outcomes, with 80% of eyes achieving a BLCVA of ≥ 0.4 logMAR. Patients with keratoconus (85%) and post-refractive surgery status (100%) exhibited best visual improvement. The mean daily wear time was 12.1 ± 2.2 h. High comfort levels were reported in 26 eyes, while 12.5% of patients discontinued using the MSDs. No significant adverse events were observed.

**Conclusion:**

MSDs represent a valuable therapeutic option offering improved visual outcomes, high levels of patient satisfaction and comfort in patients with various corneal disorders. These are a good alternative for eyes not amenable to correction with glasses or contact lenses, especially in patients planned for or awaiting corneal transplant.

## Introduction

Corneal irregularities are a significant cause of visual impairment, impacting patients’ quality of life and presenting complex challenges for ophthalmologists. Conventional treatments, such as spectacles and standard contact lenses, often fail to provide adequate correction for severe corneal disorders. In such cases, mini-scleral contact lenses or mini-scleral devices (MSDs) offer an advanced and effective alternative [1–3]. MSDs, which range from 15.4 to 18.0 mm in diameter [4], vault over the cornea and rest on the scleral tissue, creating a tear-filled reservoir between the lens and the corneal surface. This design allows MSDs to mask corneal irregularities, neutralize astigmatism, and reduce higher-order aberrations [5, 6], ultimately enhancing both visual acuity and comfort [7].

Fitting MSDs requires expertise due to their unique design and geometry. Thorough assessment of corneal topography, and tear film dynamics is required to determine the appropriate lens parameters [8]. Advanced diagnostic technologies like anterior segment optical coherence tomography (AS-OCT) and corneal tomography provide valuable insights into corneal shape, elevation, and irregularities. Specialized fitting sets with diagnostic lenses and empirical fitting algorithms based on patient demographics can also prove to be beneficial [9].

While existing literature has established the efficacy of MSDs in keratoconus, corneal scarring, and post-surgical corneal irregularities, there remains a paucity of prospective studies evaluating MSD outcomes specifically in the Indian population, where unique anatomical features such as smaller palpebral apertures and tighter eyelid tension could significantly impact lens performance and patient tolerance [10]. Furthermore, while MSDs have shown promising results in terms of both objective visual outcomes and subjective patient satisfaction, gaps remain in understanding their full potential in diverse populations.

This study aims to address these lacunae by investigating the efficacy, patient satisfaction, and visual rehabilitation outcomes associated with MSD use among Indian patients with various corneal disorders. By focusing on a population that has been underrepresented in the literature, this study seeks to provide valuable insights into the clinical benefits and challenges of MSDs, ultimately contributing to improved patient care and the development of tailored treatment strategies.

## Materials and methods

### Study design

This was an analytical, observational, prospective study designed to evaluate the efficacy of mini-scleral devices (MSDs) in patients with various corneal disorders over a period of 3 months. This was a single-center study conducted at the Department of Ophthalmology, Command Hospital (Central Command), Lucknow, India, according to tenets of the Declaration of Helsinki and the Institutional Ethics Committee (Approval No: Cert No. 021/2024, IEC Registration No: EC/NEW/INST/2021/2471). Written informed consent was obtained, from all participants or from the parents or legal guardians of any participant under the age of 16, prior to their inclusion in the study. The study followed a pre-post observational design, where each participant served as their own control.

### Study participants

This study included 32 patients (40 eyes) who were intolerant to rigid gas permeable (RGP) or soft contact lenses and had inadequate spectacle-corrected or habitually corrected visual acuity (HCVA). Demographic information, including age and sex, was collected for all participants. Detailed ocular history regarding the primary diagnosis (e.g., keratoconus, corneal scarring, post-keratoplasty status, post-refractive surgery) was documented. Systemic medical history was reviewed to screen for exclusion criteria, including active ocular infections, uncontrolled systemic diseases, or endothelial dysfunction. However, systemic variables were not analyzed separately for their potential confounding effect on visual or comfort outcomes. Patients with glaucoma, infectious keratitis, or endothelial cell dysfunction were also excluded from the study.

### Data collection

Comprehensive ophthalmic evaluations were conducted for all participants throughout the study. Initial assessments included measurements of uncorrected distance visual acuity (UCVA), HCVA prior to MSD fitting, and best lens-corrected visual acuity (BLCVA) after MSD fitting. Corneal topography was assessed via the Sirius Scheimpflug tomographer for consistent and accurate measurements. Slit-lamp examinations were conducted at each visit to monitor the fit of the MSDs and identify any complications or adverse events. Refraction was performed over the scleral lens to determine the appropriate lens power, and final prescriptions were issued two weeks after the initial trial lens fitting.

The first follow-up was scheduled one month after the fitting, whereas the second follow-up occurred at three months. A follow-up duration of three months was selected to assess early improvements in visual acuity, initial lens comfort, and daily wear patterns, as significant adaptation and stabilization typically occur within this period following mini-scleral lens fitting. During these follow-up visits, improvements in visual acuity, subjective comfort levels (measured via a five-point Likert scale), daily wear time, and the success rate for MSD acceptance were recorded. Any complications, reasons for discontinuation, or adverse events were also documented. To reduce confounding factors and ensure consistency, several strategies have been implemented. A single optometrist performed all MSD fittings for all patients. Additionally, each patient was fitted with the same brand of mini-scleral lenses (Keracare Scleral Lens), and the corneal topography of all the participants was assessed via the Sirius Scheimpflug tomographer.

### Outcome measurements

The primary outcome measures for this study included an improvement in BLCVA by ≥ 2 lines compared with HCVA, as well as the ability to wear contact lenses for ≥ 10 h per day. The secondary outcome measures focused on patient comfort, with a subjective comfort score of > 3 on a five-point Likert scale (where 1 indicates “highly uncomfortable” and 5 indicates “very comfortable”), which is considered a successful outcome. Additionally, lens acceptance was evaluated by monitoring the rate of acceptance and any reasons for discontinuation. Complications such as improper lens fit, discomfort, and any adverse events, including eye irritation or infections, were also tracked throughout the study.

### Statistical analysis

The sample size was calculated based on detecting an expected improvement in BLCVA by at least two lines on the logMAR scale.

$${\rm{Sample}}{\mkern 1mu} \,{\rm{size}},{\mkern 1mu} \,n = \frac{{{{\left( {{z_{1 - {\alpha \mathord{\left/{\vphantom {\alpha 2}} \right.\kern-\nulldelimiterspace} 2}}} + {z_{1 - \beta }}} \right)}^2} \times 2pq}}{{{{({p_1} - {p_0})}^2}}}$$

$$\:{Z}_{1-\raisebox{1ex}{$\alpha\:$}\!\left/\:\!\raisebox{-1ex}{$2$}\right.}$$ is value of α error taken at 5% and is 1.96. $$\:{Z}_{1-\beta\:}$$ is the value of β error taken at 20% (0.2) and is 0.84. Based on these assumptions and data from previously published studies including Barnett M et al., a minimum of 36 eyes was required. Anticipating possible dropouts or lens intolerance, we enrolled 40 eyes to ensure sufficient statistical power.

The logarithm of the minimum angle of resolution (logMAR) was used to compare visual acuity outcomes. The data were analyzed via SPSS Version 24.0 (SPSS, Inc., Chicago, IL, USA). Normality of continuous variables (e.g., visual acuity measurements) was assessed using the Shapiro–Wilk test. As the data were normally distributed, parametric tests (paired and unpaired t-tests) were applied for statistical comparisons. Descriptive statistics were employed to summarize patient characteristics and clinical outcomes. A p value of < 0.05 considered statistically significant.

## Results

### Study participants

**Fig. 1:**
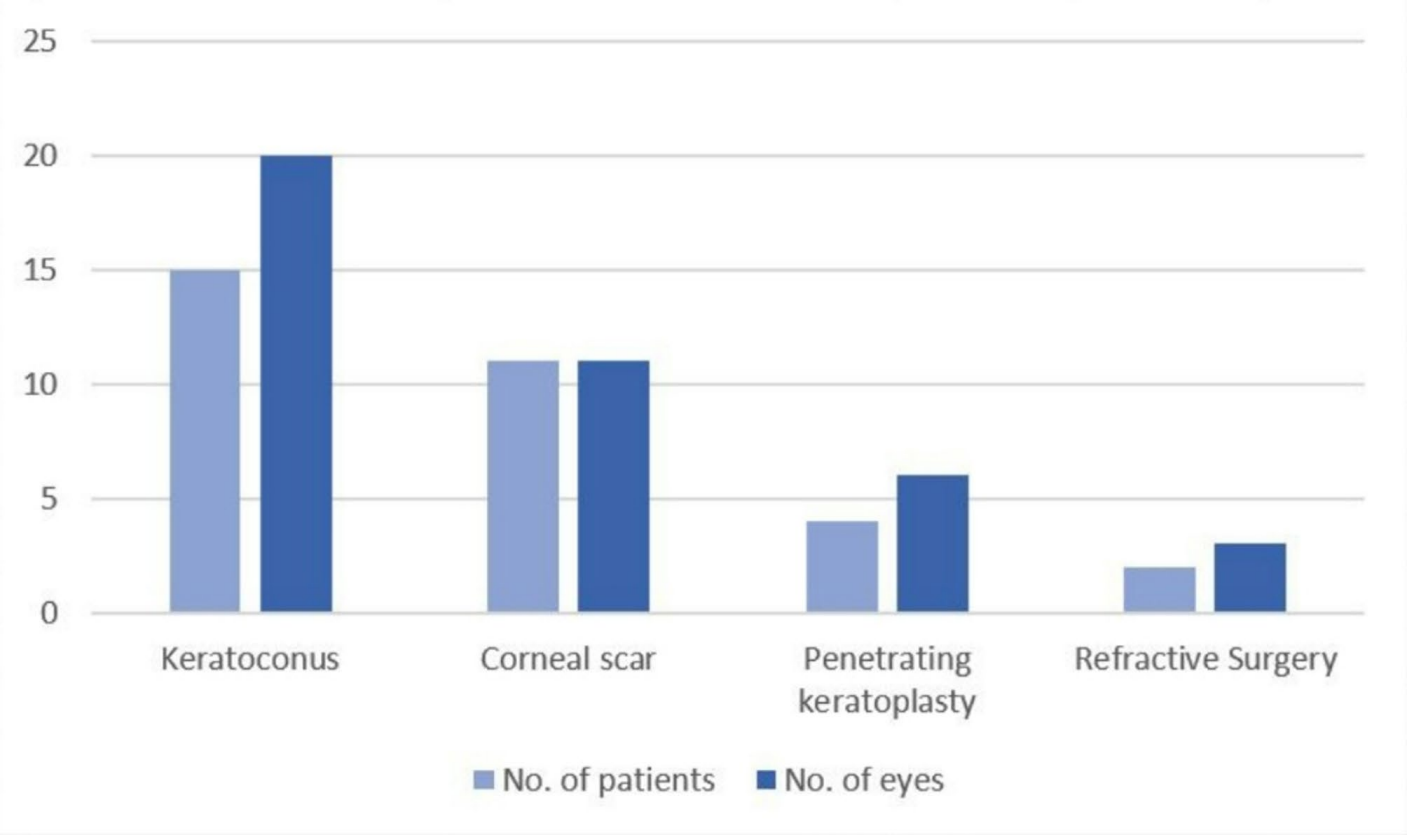
Group wise distribution of study participants

The study included 40 eyes from 32 patients (16 men and 16 women), all of whom were fitted with mini-scleral devices (MSDs). The mean age of the participants was 32.41 ± 13.62 years, with an age range of 15–68 years. The baseline uncorrected visual acuity (UCVA) was 1.07 ± 0.36 logMAR (range 0.30–1.70 logMAR). Keratoconus was the most common diagnosis, affecting 20 eyes (50.0%). Other indications included corneal scarring in 11 eyes (27.5%) due to corneal laceration, keratitis, chemical burns, ocular graft-versus-host disease, and pseudophakic bullous keratopathy. Six eyes (15.0%) had undergone corneal transplantation, and three eyes (7.5%) had post-refractive surgery status (Fig. 1). The detailed demographic data and baseline characteristics of the study participants are presented in Table 1.

**Table 1:** Baseline demographic data of the study participants

S No	Demographic	No. of patients / (%age)	No. of eyes / (%age)	Age in yr / (Range)	Male	Female	Rt Eye	Lt Eye	UCVA logMAR / (Range)
1	Keratoconus	15 (46.9)	20 (50.0)	26.20 ± 6.40 (15–36)	6	9	11	9	1.0 ± 0.34 (0.30–1.60)
2	Corneal scar	11 (34.4)	11 (27.5)	33.36 ± 12.52 (18–48)	6	5	7	4	1.2 ± 0.33 (0.40–1.70)
	Corneal laceration	5	5	36.80 ± 13.05 (19–48)	2	3	2	3	1.1 ± 0.47 (0.40–1.70)
	Keratitis	3	3	19.66 ± 2.08 (18–22)	2	1	2	1	1.3 ± 0.10 (1.20–1.40)
	Chemical burn	1	1	35	1	0	1	0	1.30
	GVHD	1	1	43	0	1	1	0	1.00
	PBK	1	1	46	1	0	1	0	1.50
3	Penetrating keratoplasty	4 (12.5)	6 (15.0)	58.00 ± 7.48 (50–68)	2	2	3	3	1.3 ± 0.18 (1.10–1.60)
4	Refractive Surgery	2 (6.3)	3 (7.5)	22.50 ± 6.36 (18–27)	2	0	1	2	0.60 ± 0.36 (0.30–1.0)
	**Total**	**32**	**40**	**32.41 ± 13.62 (15–68)**	**16**	**16**	**22**	**18**	**1.07 ± 0.36 (0.30–1.70)**

Values are presented as mean ± standard deviation. UCVA = uncorrected distance visual acuity;

logMAR = logarithm of the minimum angle of resolution

### Corneal topography and lens fitting parameters

Corneal topographic indices, white-to-white distance, central corneal thickness (CCT), intraocular pressure (IOP), and lens fitting parameters were compared between the study participants (Table 2). Patients with post-refractive surgery status presented flatter keratometry (K_flat = 38.2 ± 3.5 D; K_steep = 40.6 ± 2.65 D) values than did the other groups. In contrast, the keratoconus group presented steeper K values (K_flat = 44.5 ± 1.71 D; K_steep = 50.6 ± 1.85 D). The K values among patients with corneal scarring and post-penetrating keratoplasty were variable. Across the entire study population, the mean power of the MSDs was − 3.28 ± 3.42 D, with a mean base curve of 7.42 ± 0.08 mm and a mean diameter of 16.09 ± 0.50 mm.

**Table 2:** Comparative data for corneal topographic indices

Variable	Keratoconus (*n* = 20)	Corneal Scar (*n* = 11)	Penetrating Keratoplasty (*n* = 6)	Refractive Surgery (*n* = 3)	Total (*n* = 40)
**Topography (D)**					
*Flat K*	44.5 ± 1.71	40.1 ± 2.04	42.2 ± 2.42	38.2 ± 3.5	42.5 ± 2.99
*Steep K*	50.6 ± 1.85	47.8 ± 2.19	49.6 ± 2.28	40.6 ± 2.65	48.9 ± 3.34
*Astigmatism*	6.09 ± 1.71	7.68 ± 3.63	7.4 ± 1.89	2.4 ± 0.90	6.4 ± 2.68
**White-to-white (mm)**	12.0 ± 0.19	11.3 ± 0.39	11.8 ± 0.23	11.6 ± 0.17	11.75 ± 0.39
**CCT (µm)**	457.2 ± 55.4	495.5 ± 50.7	584.5 ± 59.9	472.7 ± 34.6	488 ± 68.04
**IOP (mmHg)**	14.5 ± 3.33	15.2 ± 1.94	13.0 ± 3.74	14.3 ± 4.0	14.45 ± 3.08
**Lens fitting parameters**					
*(a) Base curve (mm)*	7.42 ± 0.08	7.43 ± 0.08	7.39 ± 0.08	7.45 ± 0.09	7.42 ± 0.08
*(b) Power (D)*	–5.26 ± 2.81	–2.48 ± 3.56	–4.83 ± 5.43	–1.22 ± 2.54	–3.28 ± 3.42
*(c) Diameter (mm)*	16.23 ± 0.52	15.8 ± 0.38	15.98 ± 0.49	16.4 ± 0.50	16.09 ± 0.50

Values are presented as mean ± standard deviation. CCT = Central corneal thickness; IOP = Intra Ocular Pressure

### Primary outcomes

**Fig. 2:**
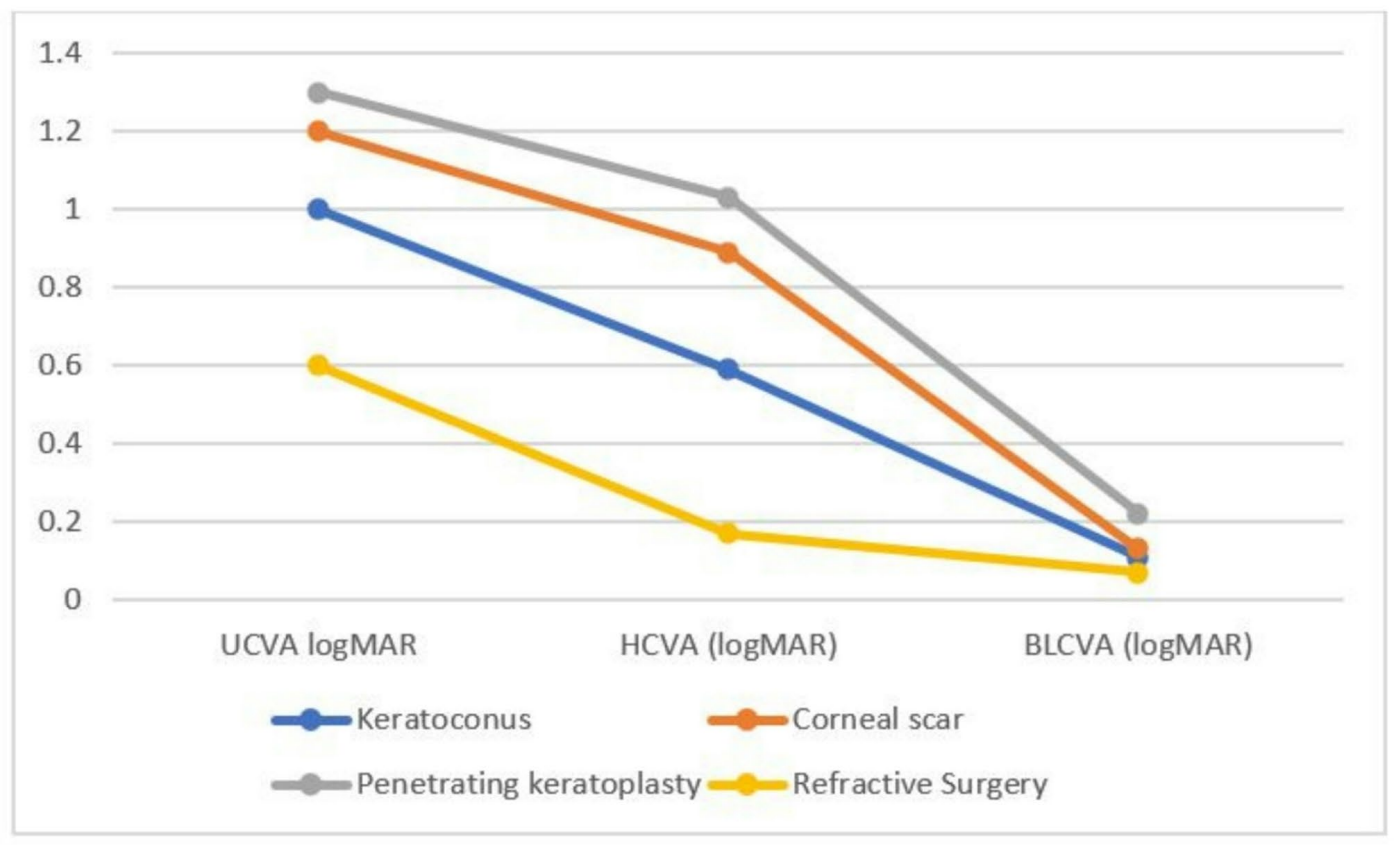
Comparison of UCVA, HCVA, BLCVA between study groups

The mean UCVA before MSD fitting was 1.07 ± 0.36 logMAR (range 0.30 to 1.70 logMAR), and the mean HCVA was 0.71 ± 0.39 logMAR (range 0.0 to 1.70 logMAR) (Fig. 2). The differences between the UCVA and HCVA were statistically significant in the keratoconus group (*p* = 0.026) but not in the corneal scar (*p* = 0.138), post-penetrating keratoplasty (*p* = 0.147), or post-refractive surgery (*p* = 0.378) groups.

Following scleral lens fitting, the BLCVA improved to 0.16 ± 0.20 logMAR (range 0.0 to 0.6 logMAR). The difference between BLCVA and HCVA was statistically significant in patients with keratoconus (*p* = 0.005), corneal scarring (*p* = 0.033), and post-penetrating keratoplasty status (*p* = 0.0026) but not in patients with post-refractive surgery status (*p* = 0.114) (Table 3).

**Fig. 3:**
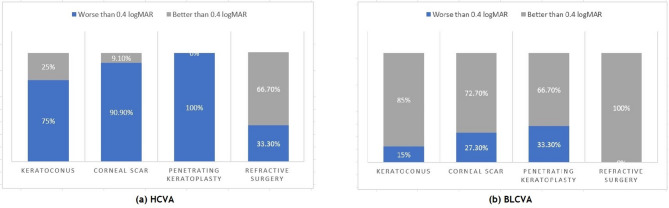
Comparison of visual acuity before (HCVA) and after scleral lens fitting (BLCVA)

Among the 40 eyes, 32 achieved a BLCVA of ≥ 0.4 logMAR following scleral lens fitting. Eight eyes had a BLCVA of < 0.4 logMAR. Patients with post-refractive surgery status and keratoconus had the best BLCVA results, with 100% and 85% of eyes, respectively, achieving a BLCVA ≥ 0.4 logMAR (Table 4; Fig. 3).

**Table 3:** UCVA, HCVA, BLCVA, and differences between UCVA and HCVA, and BLCVA and HCVA

Variable	Keratoconus (*n* = 20)	Corneal Scar (*n* = 11)	Penetrating Keratoplasty (*n* = 6)	Refractive Surgery (*n* = 3)	Total (*n* = 40)
UCVA (logMAR)	1.0 ± 0.34	1.2 ± 0.33	1.3 ± 0.18	0.60 ± 0.36	1.07 ± 0.36
HCVA (logMAR)	0.59 ± 0.30	0.89 ± 0.40	1.03 ± 0.21	0.17 ± 0.29	0.71 ± 0.39
BLCVA (logMAR)	0.15 ± 0.18	0.16 ± 0.22	0.23 ± 0.26	0.07 ± 0.12	0.16 ± 0.20
Difference between UCVA and HCVA (logMAR); *p*-value	0.41 ± 0.12 **0.026**	0.31 ± 0.19 0.138	0.27 ± 0.14 0.147	0.43 ± 0.12 0.378	0.36 ± 0.15
Difference between BLCVA and HCVA (logMAR); *p*-value	0.44 ± 0.17 **0.005**	0.72 ± 0.25 **0.033**	0.80 ± 0.11 **0.0026**	0.10 ± 0.17 0.114	0.55 ± 0.27

Values are presented as mean ± standard deviation. UCVA = uncorrected distance visual acuity;

HCVA = habitually corrected distance visual acuity; BLCVA = best lens-corrected visual acuity with scleral lenses; logMAR = logarithm of the minimum angle of resolution. Statistical test used: Paired t-test

In the post-penetrating keratoplasty group, although 66.7% of eyes achieved a BLCVA ≥ 0.4 logMAR, some eyes did not achieve optimal visual acuity. The suboptimal outcomes in these eyes were likely due to significant irregular astigmatism, higher-order aberrations, or corneal graft-host junction irregularities that could not be fully neutralized even with MSDs.

**Table 4:** Proportions of eyes achieving better or worse than 0.4 LogMAR visual acuity before and after Mini-Scleral Lens fitting

Visual Acuity	Keratoconus (*n* = 20)		Corneal Scar (*n* = 11)		Penetrating Keratoplasty (*n* = 6)		Refractive Surgery (*n* = 3)		Total (*n* = 40)	
	HCVA	BLCVA	HCVA	BLCVA	HCVA	BLCVA	HCVA	BLCVA	HCVA	BLCVA
**Worse than 0.4 logMAR**	15 (75%)	3 (15%)	10 (90.9%)	3 (27.3%)	6 (100%)	2 (33.3%)	1 (33.3%)	0 (0%)	32 (80%)	8 (20%)
**Better than 0.4 logMAR**	5 (25%)	17 **(85%)**	1 (9.10%)	8 (72.7%)	0 (0%)	4 (66.7%)	2 (66.7%)	3 **(100%)**	8 (20%)	32 (80%)

HCVA = habitually corrected distance visual acuity; BLCVA = best lens-corrected visual acuity with scleral lenses; logMAR = logarithm of the minimum angle of resolution. A visual acuity of 0.4 logMAR equals to Snellen’s 80/200

The mean daily wear time of the scleral lenses was 12.1 ± 2.2 h among the 35 eyes of 28 patients who continued wearing MSDs at 3-month follow-up

### Secondary outcomes

Subjective comfort on a five-level Likert scale was studied, and MSDs were reportedly comfortable or very comfortable in 26 eyes and neither uncomfortable nor comfortable in 9 eyes. At the end of the 3-month follow-up, 4 patients (12.5%; or 5 eyes, 12.5%) discontinued the use of MSDs. We compared the profiles of patients who reported high comfort levels versus those who were intolerant or had equivocal responses. Patients who discontinued MSD use or reported lower comfort levels tended to have steeper mean keratometry readings (average K > 48 D) and more extensive corneal scarring. No significant adverse events were reported by the study participants during the follow-up period.

## Discussion

The therapeutic applications of MSDs have been steadily increasing with the advent of advanced diagnostic technologies such as AS-OCT and corneal tomography. Despite these advancements, there is limited research evaluating both objective measures (visual acuity and corneal topography) and subjective factors (comfort and ease of wear) among Indian patients with corneal disorders. Anatomical differences, such as smaller palpebral fissures and tighter eyelids in the Indian population, could influence the tolerability and performance of MSDs. Our study provides novel insights into this underrepresented demographic.

Our study demonstrated that MSDs significantly improved visual outcomes, with 80% of eyes (32 out of 40) achieving a best lens-corrected visual acuity (BLCVA) of ≥ 0.4 logMAR. Specifically, patients with post-refractive surgery status (100%) and keratoconus (85%) had the best outcomes. Additionally, MSDs were well tolerated, with most patients reporting high comfort levels, and the average daily wear time was 12.1 ± 2.2 h. Despite a small percentage of patients discontinuing use (12.5%), no significant adverse events were observed. These findings underscore the potential of MSDs as effective options for improving both visual acuity and comfort in patients with corneal disorders.

In patients with keratoconus, previous studies have reported significant improvements in visual acuity and quality of life with MSDs [11–13]. A study by Kreps EO et al. revealed significant improvements in visual acuity and visual functioning with the use of MSDs in patients with keratoconus [11]. Our study corroborates these findings, demonstrating substantial visual gains in the keratoconus group. Visual acuity improved to a greater extent with the use of MSDs, as 17 eyes (85%) achieved a BLCVA of 0.4 logMAR or better than did only 5 eyes (25%) before the use of these lenses. The few keratoconus cases that did not achieve BLCVA ≥ 0.4 logMAR were complicated by stromal scarring or intracorneal ring segments, which may have disrupted the regularity of the tear reservoir needed for optimal optics.

Among patients with corneal scarring, 72.7% achieved BLCVA ≥ 0.4 logMAR. The flatter and more regular shape of scarred corneas, compared to severely ectatic corneas, may have contributed to effective tear reservoir formation and better outcomes. Similar trends have been reported in prior studies evaluating MSDs in corneal scarring cases.

Post-keratoplasty patients achieved favourable visual outcomes, with 66.7% attaining BLCVA ≥ 0.4 logMAR. In a study conducted by Barnett M et al., 44 eyes (91.7%) achieved functional vision with a BLCVA of 0.3 logMAR or better following the use of MSDs in eyes after keratoplasty due to progressive keratoconus [14]. The slightly lower success rate in our study may reflect the complex corneal surfaces resulting from varied indications for keratoplasty, including traumatic injuries and infections.

In post-refractive surgery patients, MSDs offered excellent visual rehabilitation, with all patients achieving BLCVA ≥ 0.4 logMAR. This underscores the potential role of MSDs in addressing irregular astigmatism and glare symptoms following procedures like radial keratotomy and LASIK. Chu et al. [15] revealed that a uniform fluid reservoir under the scleral lens significantly affects visual outcomes in correcting irregular astigmatism after radial keratotomy. The vergence of light across the cornea is increased as the scleral lens completely vaults the cornea and creates a fluid reservoir [16]. Few studies have reported that symptoms caused by heterogeneous and dispersed or disseminated ablation during refractive surgery do not completely subside even with the use of MSDs [17].

In our study, patients with keratoconus presented steeper K values (K_flat_ = 44.5 ± 1.71 D and K_steep_ = 50.6 ± 1.85 D), and post-refractive surgery patients presented flatter K values (K_flat_ = 38.2 ± 3.5 D and K_steep_ = 40.6 ± 2.65 D) than did patients in the other disease groups. These two groups eventually had the best BLCVA, with 100% and 85% of the eyes having visual acuity ≥ 0.4 logMAR for the post-refractive surgery status and keratoconus groups, respectively. In patients with post-penetrating keratoplasty status and corneal scarring, the difference between the UCVA and HCVA was the smallest and statistically insignificant; however, among patients in these two groups, only the difference between the HCVA and BLCVA was the greatest and statistically significant. This finding further suggests that the lower the HCVA is, the greater the difference between the HCVA and BLCVA or improvement in visual acuity with MSDs.

Despite its strengths, our study has several limitations. The main limitations were the relatively small sample size and the short follow-up period. Additionally, because the study included patients with a variety of corneal disorders, the representation of each specific condition was limited, making it difficult to draw definitive conclusions for each disease group. As a result, the full effect of MSDs on individual corneal conditions could not be thoroughly studied. Although a pre-post comparison within the same subjects reduces inter-individual variability, another limitation of this study was the absence of an external control group. Future studies with larger sample sizes and longer follow-up periods are needed to provide more comprehensive insights into the effectiveness of MSDs in patients with corneal diseases, particularly in the Indian subcontinent.

An important contribution of our study is the evaluation of MSDs in an Indian population. Given the anatomical and demographic differences compared to Western populations, our findings offer valuable insights that could inform fitting strategies and enhance visual rehabilitation outcomes for Indian patients with corneal disorders.

## Conclusion

MSDs represent a valuable therapeutic option for patients with corneal disorders, offering improved visual outcomes, enhanced comfort, and high levels of patient satisfaction. These lenses are a good alternative for eyes that are not amenable to correction with glasses, soft contact lenses or RGP, especially in patients with keratoconus or corneal scars that are being planned for corneal transplant. Continued research and innovation in this field are essential to further optimize lens design, fitting techniques, and treatment outcomes, ultimately improving the lives of individuals with corneal disorders.

## Data Availability

The datasets generated and/or analysed during the current study are not publicly available due to institutional protocols and norms, but are available from the corresponding author on reasonable request.

## Abbreviations

MSD: Mini Scleral Device(s)
AS-OCT: Anterior Segment Optical Coherence Tomography
PBK: Pseudophakic Bullous Keratopathy
GVHD: Graft-Versus-Host Disease
UCVA: Uncorrected distance visual acuity
HCVA: Spectacle corrected or habitually corrected visual acuity
BLCVA: Best lens corrected visual acuity
LogMAR: Logarithm of the Minimum Angle of Resolution
CCT: Central Corneal Thickness
IOP: Intraocular pressure
